# Association of Highly Restrictive State Abortion Policies With Abortion Rates, 2000-2014

**DOI:** 10.1001/jamanetworkopen.2020.24610

**Published:** 2020-11-09

**Authors:** Benjamin P. Brown, Luciana E. Hebert, Melissa Gilliam, Robert Kaestner

**Affiliations:** 1 Division of General Obstetrics and Gynecology, Alpert Medical School of Brown University, Women and Infants Hospital of Rhode Island, Providence; 2 Institute for Research and Education to Advance Community Health, Elson S. Floyd College of Medicine, Washington State University, Seattle; 3 Departments of Obstetrics and Gynecology and Pediatrics, University of Chicago, Illinois; 4 Harris School of Public Policy, University of Chicago, Illinois

## Abstract

**Importance:**

Although abortion is common in the United States, patients face substantial barriers to obtaining an abortion. Recently enacted abortion restrictions pose such barriers.

**Objectives:**

To assess the association between a state legislative climate that is highly restrictive toward abortion provision and the abortion rate and to evaluate whether distance to a facility providing abortion care mediates the association between legislative climate and the abortion rate.

**Design, Setting, and Participants:**

This cohort study examined county-of-residence abortion rates from all states that publicly provided them and used data on abortion restrictions, facility locations, and county demographic characteristics for the years 2000 to 2014. The association between legislative climate and abortion rates was evaluated using propensity score–weighted, linear regression difference-in-difference analysis. All models included state and year fixed effects and standard errors adjusted for state-level clustering.

**Exposures:**

Highly restrictive legislative climate, defined as having at least 3 of 4 types of abortion restrictions; distance to a high-volume facility providing abortion care (ie, performing ≥395 abortions per year) in miles.

**Main Outcomes and Measures:**

County-level abortion rate, defined as abortions per 1000 women per year.

**Results:**

Abortion rate data were obtained from 1178 counties in 18 states for a median of 12.5 years (range, 5-14). The median abortion rate was 2.89 per 1000 women (interquartile range, 1.71-4.46 per 1000 women). A highly restrictive legislative climate, when compared with a less restrictive one, was associated with 0.48 fewer abortions per 1000 women (95% CI, −0.92 to −0.04 abortions per 1000 women; *P* = .03). Adjusted for distance to a facility providing abortion care, a highly restrictive legislative climate was associated with 0.44 fewer abortions per 1000 women (95% CI, −0.85 to −0.03; *P* = .04). Each mile to a facility was associated with 0.02 fewer abortions per 1000 women (95% CI, −0.03 to −0.01 abortions per 1000 women; *P* = .003). Legislative climate was not significantly associated with distance to a facility providing abortion care (change in distance associated with highly restrictive climate, −2.73 [95% CI, −6.02 to 0.57] miles; *P* = .10).

**Conclusions and Relevance:**

This study provides evidence that a state legislative climate that is highly restrictive toward abortion provision is associated with a lower abortion rate. The cumulative effect of restrictive policies may pose a barrier to abortion access.

## Key Points

**Question:**

Is a highly restrictive state policy climate associated with a lower abortion rate?

**Findings:**

This cohort study of 1178 counties in 18 states evaluated the association between a highly restrictive state legislative climate and the abortion rate in 18 states between 2000 and 2014. A highly restrictive policy climate, when compared with a less restrictive one, was associated with a significantly lower abortion rate by 0.48 abortions per 1000 women, representing a 17% decrease from the median abortion rate of 2.89 per 1000 women.

**Meaning:**

This study provides evidence that a highly restrictive state legislative climate is associated with a lower abortion rate.

## Teaser

This cohort study evaluates the association of restrictive abortion laws in 18 states with state abortion rates.

## Introduction

Although abortion is common in the US,^1^ patients must frequently surmount substantial barriers to obtain timely abortion care. State legislative restrictions—hundreds of which have been enacted by states in recent years—represent such a barrier.^2,3,4^ Some laws directly impede individuals from accessing care, such as those that require multiple trips to a facility providing abortion care to obtain an abortion. Others, known as targeted regulations of abortion providers (TRAP laws), and which include facility requirements such as corridor widths or ceiling heights, may be impossible for facilities to implement because of cost or space constraints. By changing how or where services are available, such restrictions may make it more difficult for patients to access abortion.^5,6,7^

Restrictions on sexual health care have historically taken many forms, including prohibitions on contraception, requirements for spousal or parental notification before abortion, and restrictions on abortion facility funding. Much of the evidence demonstrating the burden posed by contemporary abortion restrictions cites the experience in Texas after the passage of HB2, which imposed ambulatory surgical center requirements on all abortion providing facilities and mandated that clinicians providing abortions have hospital admitting privileges. These restrictions resulted in statewide clinic closures. The law was associated with longer travel distances and higher out-of-pocket costs for abortion patients^5^; in Texas counties with travel distances of 50 km or more to a facility, the number of abortions declined by 20% and the number of births increased by 3% following the passage of HB2.^8^ Earlier data from Texas suggest that some patients were ultimately able to access abortion out of state after a 2004 law reduced the availability of second trimester abortion,^7^ but data on the influence of parental involvement laws suggest that out-of-state travel does not fully account for the decrease in in-state abortions after implementation of such policies.^9^ Moreover, out-of-state travel requires increased investment of time and money, which can pose an access barrier.

Some studies have also examined the influence of restrictive policies across multiple states. Hawkins et al^10^ found that gestational age-based abortion restrictions were associated with a 38% increase in maternal mortality, and a 20% reduction in the number of Planned Parenthood clinics was associated with an 8% increase in maternal mortality. Austin and Harper^11^ performed a difference-in-difference analysis to evaluate the influence of ambulatory surgical center and admitting privileges requirements and did not find a significant association between these laws and the abortion rate.

In this study, we sought to expand the literature in 2 key ways: by using multistate, longitudinal data to compare abortion legislation and abortion rates over time and by evaluating the association of an overall restrictive legislative climate with abortion rates. To do so, we constructed a restrictiveness variable using 4 common categories of abortion restrictions: delays between counseling and abortion, parental involvement in a minor’s abortion, TRAP laws, and gestational age cutoffs. We estimated the change in the abortion rate associated with a highly restrictive climate by performing a propensity score–weighted, linear regression difference-in-difference analysis. We also performed secondary analyses to evaluate whether distance to a facility providing abortion care mediates the association between a highly restrictive policy climate and the abortion rate.

## Methods

### Study Design

We used a propensity score–weighted difference-in-difference design with a linear regression model to estimate the association between a highly restrictive state legislative climate and the abortion rate, in comparison with a less restrictive climate. In some models, we included only states that became highly restrictive during our study to ensure our findings were not primarily a reflection of underlying differences between states that were never highly restrictive and those that were. In other models, we included adjustment for distance to a facility providing abortion care. Additionally, we performed a set of secondary analyses to examine whether distance to a facility was associated with enforcement of a highly restrictive legislative climate.

### Setting and Participants

To generate our data set, we linked abortion rates, legislative data, and facility locations for the years 2000 to 2014. Because we included distance to a facility and because we sought to evaluate the influence of a state’s laws on its residents, we required county-of-residence abortion data. We collected data from the vital statistics offices of all states that publicly provided such information (Table 1). Because only 1 state provided abortion data for 2000, we dropped this year from our analysis of the association of state policy with abortion rates. Mean county-level demographic characteristics of states that provided county-of-residence abortion data and those that did not are summarized in Table 2. Variables were compared with *t* tests.

**Table 1.** Number of Counties in State Vital Statistics Abortion Data Included in Analyses by State and Year, With Years Under a Highly Restrictive Legislative Climate Highlighted

State (total counties, No.)	Counties with data included in analysis, No. (%)													
	2001	2002	2003	2004	2005	2006	2007	2008	2009	2010	2011	2012	2013	2014
Arizona (n = 15)	10 (66.7)	10 (66.7)	10 (66.7)	10 (66.7)	10 (66.7)	10 (66.7)	10 (66.7)	10 (66.7)	10 (66.7)	15 (100)^a^	NR^a^	NR^a^	NR^a^	NR^a^
Delaware (n = 3)	3 (100)	3 (100)	3 (100)	3 (100)	3 (100)	3 (100)	3 (100)	3 (100)	NR	NR	NR	NR^a^	NR^a^	NR^a^
Georgia (n = 159)	30 (18.9)	30 (18.9)	30 (18.9)	30 (18.9)	30 (18.9)	30 (18.9)^a^	32 (20.1)^a^	32 (20.1)^a^	32 (20.1)^a^	159 (100)^a^	NR^a^	NR^a^	NR^a^	NR^a^
Illinois (n = 102)	NR	NR	NR	NR	21 (20.6)	21 (20.6)	21 (20.6)	21 (20.6)	22 (21.6)	26 (25.5)	26 (25.5)	27 (26.5)	26 (25.5)	26 (25.5)^a^
Indiana (n = 92)	24 (26.1)^a^	24 (26.1)^a^	24 (26.1)^a^	24 (26.1)^a^	24 (26.1)^a^	25 (27.2)^a^	25 (27.2)^a^	25 (27.2)^a^	25 (27.2)^a^	82 (89.1)^a^	87 (94.6)^a^	86 (93.5)^a^	80 (87.0)^a^	92 (100)^a^
Michigan (n = 83)	NR^a^	NR^a^	NR^a^	NR^a^	NR^a^	28 (33.7)^a^	29 (34.9)^a^	29 (34.9)^a^	29 (34.9)^a^	78 (94.0)^a^	83 (100)^a^	83 (100)^a^	83 (100)^a^	83 (100)^a^
New York (n = 62)	38 (61.3)	38 (61.3)	38 (61.3)	38 (61.3)	38 (61.3)	38 (61.3)	38 (61.3)	38 (61.3)	38 (61.3)	62 (100)	62 (100)	62 (100)	62 (100)	62 (100)
North Carolina (n = 100)	37 (37.0)	37 (37.0)	37 (37.0)	37 (37.0)	37 (37.0)	37 (37.0)	37 (37.0)	37 (37.0)	37 (37.0)	100 (100)	100 (100)	100 (100)^a^	100 (100)^a^	100 (100)^a^
Ohio (n = 88)	38 (43.2)^a^	38 (43.2)^a^	38 (43.2)^a^	38 (43.2)^a^	38 (43.2)^a^	38 (43.2)^a^	38 (43.2)^a^	38 (43.2)^a^	38 (43.2)^a^	88 (100)^a^	88 (100)^a^	88 (100)^a^	88 (100)^a^	88 (100)^a^
Oklahoma (n = 77)	NR	10 (13.0)	10 (13.0)	10 (13.0)	10 (13.0)	11 (14.3)^a^	NR^a^	NR^a^	NR^a^	NR^a^	NR^a^	NR^a^	NR^a^	NR^a^
Oregon (n = 36)	15 (41.7)	15 (41.7)	15 (41.7)	15 (41.7)	15 (41.7)	15 (41.7)	15 (41.7)	15 (41.7)	15 (41.7)	35 (97.2)	36 (100)	36 (100)	36 (100)	36 (100)
Pennsylvania (n = 67)	38 (56.7)^a^	38 (56.7)^a^	38 (56.7)^a^	38 (56.7)^a^	38 (56.7)^a^	39 (58.2)^a^	39 (58.2)^a^	39 (58.2)^a^	39 (58.2)^a^	67 (100)^a^	67 (100)^a^	67 (100)^a^	67 (100)^a^	67 (100)^a^
South Carolina (n = 46)	20 (43.5)^a^	20 (43.5)^a^	20 (43.5)^a^	20 (43.5)^a^	20 (43.5)^a^	20 (43.5)^a^	21 (45.7)^a^	21 (45.7)^a^	21 (45.7)^a^	46 (100)^a^	46 (100)^a^	46 (100)^a^	46 (100)^a^	46 (100)^a^
Texas (n = 254)	NR	NR	NR	NR^a^	NR^a^	50 (19.7)^a^	50 (19.7)^a^	50 (19.7)^a^	50 (19.7)^a^	254 (100)^a^	254 (100)^a^	254 (100)^a^	254 (100)^a^	254 (100)^a^
Utah (n = 29)	NR^a^	NR^a^	NR^a^	NR^a^	NR^a^	NR^a^	6 (20.7)^a^	6 (20.7)^a^	6 (20.7)^a^	29 (100)^a^	29 (100)^a^	29 (100)^a^	29 (100)^a^	29 (100)^a^
Vermont (n = 14)	NR	NR	NR	NR	NR	NR	NR	NR	1 (7.1)	14 (100)	14 (100)	14 (100)	14 (100)	NR
Washington (n = 39)	18 (46.2)	18 (46.2)	18 (46.2)	18 (46.2)	18 (46.2)	19 (48.7)	19 (48.7)	19 (48.7)	19 (48.7)	39 (100)	39 (100)	39 (100)	39 (100)	39 (100)
Wisconsin (n = 72)	22 (30.6)^a^	22 (30.6)^a^	22 (30.6)^a^	22 (30.6)^a^	22 (30.6)^a^	22 (30.6)^a^	14 (19.4)^a^	16 (22.2)^a^	18 (25.0)^a^	13 (18.1)^a^	13 (18.1)^a^	14 (19.4)^a^	10 (13.9)^a^	49 (68.1)^a^

Abbreviation: NR, not reported.

^a^ Years when a highly restrictive legislative climate was in effect, defined as having 3 or more of 4 types of restrictions in state law: mandatory delays between counseling and abortion, parental involvement in a minor’s abortion, targeted regulations of facilities providing abortion care that are not imposed on other health care facilities, and gestational age cutoffs.

**Table 2.** Mean County-Level Demographic Data for States With and Without Abortion Rate Data

	States with abortion data (n = 18)	States without abortion data (n = 33)^a^	*P* value^b^
Population			
Women, No. (SD)	56 810 (33 281)	42 326 (63 989)	<.001
Race/ethnicity, %			
American Indian/Alaska Native (SD)	1.6 (2.6)	3.1 (6.7)	<.001
Asian (SD)	0.9 (0.4)	1.1 (1.9)	<.001
African American (SD)	9.3 (10.1)	8.3 (11.1)	<.001
White (SD)	83.1 (10.1)	83.5 (12.7)	<.001
Hispanic (SD)	9.2 (10.3)	5.4 (7.4)	<.001
Median income, $ (SD)	44 817 (3747)	44 016 (9593)	<.001
College graduates, % (SD)	25.1 (2.9)	28.7 (5.3)	<.001
Voting for the Democratic candidate in the most recent presidential election, No. (%; SD)	22 808 (39.4; 7.1)	17 359 (39.2; 7.5)	<.001

^a^ Including Washington, DC.

^b^ *P* values generated using *t* tests.

This study was determined to be exempt by the institutional review board of the University of Chicago Biological Sciences Division, and received a nonhuman participants research designation from the institutional review board of the Women and Infants Hospital of Rhode Island. Because this study only used county-level, deidentified data, the requirement for individual participant informed consent was waived. We followed the Strengthening the Reporting of Observational Studies in Epidemiology (STROBE) reporting guideline in reporting this study.

### Variables, Data Sources, and Measurement

The abortion rate was defined as the total number of abortions obtained by residents of a county in a given year divided by the total female population.^12^ Through a direct data-sharing agreement, we obtained archived versions of the Guttmacher Institute’s reports of laws in effect on January 1 of each year in the study period. We identified states that imposed abortion restrictions of 4 types: delays between counseling and abortion, parental involvement in a minor’s abortion, TRAP laws, and gestational age cutoffs. States that had 3 or 4 types of restrictions were considered highly restrictive. Those with fewer than 3 were considered less restrictive.^13^

To estimate distance to a facility providing abortion care, we used the geodist package in Stata, version 14 (StataCorp) to calculate straight-line distances between the population centroid of each county and that of the closest county identified by the Guttmacher Institute as having a high-volume facility (ie, a doctor or clinic performing ≥395 abortions per year).^14,15^

Propensity scores were derived from county-specific demographic data from the US Census Bureau, including the percentage of the population in each race and ethnicity category,^16,17^ median income,^12^ and total female population.^12^ The percentage who voted for the Democratic candidate in the most recent presidential election was obtained from Leip’s Atlas of Presidential Elections.^18^ We addressed data gaps in demographic variables by using the estimate from the nearest year for which an estimate was available.

### Bias

State vital statistics data are often biased due to poor reporting.^19^ The Guttmacher Census, meanwhile, is widely considered the criterion standard for US abortion incidence data but does not provide data by county of residence. Therefore, we validated our state-reported data against data from the Guttmacher Institute’s Abortion Provider Census by generating pairwise correlation coefficients by state and year.

### Statistical Analysis

Analysis was performed using Stata 14 (StataCorp). A priori significance level was *P* = .05, and hypothesis tests were 2-sided. All models were linear regression difference-in-difference estimates, employing propensity score weighting, state and year fixed effects, and standard errors were robust to state-level clustering.^20^

A difference-in-difference model usually compares 2 groups of counties that are demographically similar except for the introduction of the policy under investigation and assesses change over time in an outcome of interest (in this case, the abortion rate). If the prepolicy trends in the exposed and unexposed groups are parallel, it is plausible to conclude that deviation from a parallel trend in the exposed counties in the postpolicy period is associated with the policy itself. When a policy change occurs at a single time across jurisdictions, the parallel trends assumption can be tested by directly comparing the prepolicy trends in exposed and unexposed jurisdictions.

However, in our data, policy introduction dates were staggered. To test the parallel trends assumption, we regressed the abortion rate on dummy variables for the years before and after the introduction of highly restrictive legislation with state and year fixed effects to determine whether the abortion rate in each of these county-year observations differed systematically from the abortion rate in other county-year observations. Because we included year fixed effects, potentially confounding temporal trends were held constant across groups. Therefore, each coefficient in the plot in this study addressed the question of whether the abortion rate was significantly different in the years around adoption of a highly restrictive climate than it was in other years.

## Results

We obtained data for 1178 counties in 18 states representing a geographically diverse sample.^21,22,23,24,25,26,27,28,29,30,31,32,33,34,35,36,37,38^ These data had ranges from 5 to 14 years, and a median of 12.5 years (range, 5-14 years) (Table 1). The abortion rate had a median (interquartile range [IQR]) of 2.89 (1.71-4.46) per 1000 women. Correlation coefficients for validation between state data and those from the Guttmacher Institute were consistently greater than 0.7 (only 1 value was less, at 0.68), and most were greater than 0.9. The results of the test for violations of the parallel trend criterion were nonsignificant, suggesting the parallel trend assumption was valid (Figure).

**Figure.**
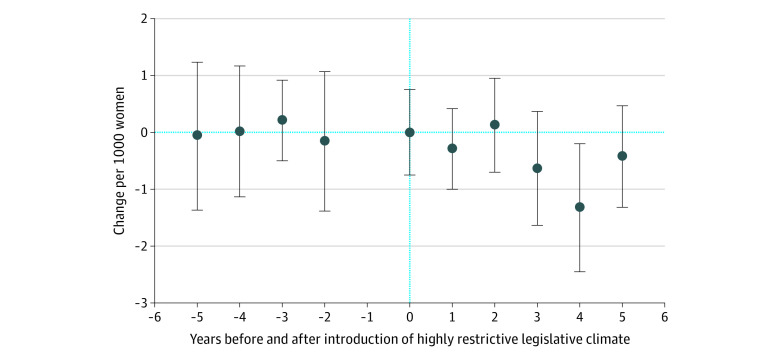
Difference in Abortions per 1000 Women Before and After Introduction of Highly Restrictive Legislative Climate Error bars indicate 95% CIs for each point estimate. A highly restrictive legislative climate was defined as a state that instituted abortion restrictions in 3 or more of 4 major categories of laws. The year before introduction of a highly restrictive climate was excluded as the baseline year. The model employed propensity score weighting, state and year fixed effects, and cluster-robust standard errors.

The main results of this study are presented in Table 3. Highly restrictive legislative climates were associated with an abortion rate decrease of 0.48 abortions per 1000 women (95% CI, −0.92 to −0.04; *P* = .03) compared with a less restrictive one. When only the 14 states that became highly restrictive during the study period were included, the highly restrictive legislative climate was associated with an abortion rate decrease of 0.45 abortions per 1000 women (95% CI, −0.80 to −0.10 abortions per 1000 women; *P* = .02). When adjusted for distance to a facility, a highly restrictive state legislative climate was associated with an abortion rate decrease of 0.44 abortions per 1000 women (95% CI, −0.85 to −0.03 abortions per 1000 women; *P* = .04). Each mile of distance to a facility was associated with an abortion rate decrease of 0.02 abortions per 1000 women (95% CI, −0.03 to −0.01 abortions per 1000 women; *P* = .003). In models assessing the association between a highly restrictive climate and distance to a facility providing abortion care, there was no statistically significant association between policy climate and distance to a facility (change in distance associated with highly restrictive climate, −2.73 [95% CI, −6.02 to 0.57] miles; *P* = .10) (Table 4).

**Table 3.** Change in Number of Abortions per 1000 Women Associated With Highly Restrictive Legislative Climate

Characteristic	Primary model (n = 8029)		Only states that became highly restrictive during study period (n = 6645)		Adjusted for distance (n = 5379)^a^	
	β (95% CI)	*P* value	β (95% CI)	*P* value	β (95% CI)	*P* value
Highly restrictive legislative climate	−0.48 (−0.92 to −0.04)	.03	−0.45 (−0.80 to −0.10)	.02	−0.44 (−0.85 to −0.03)	.04
Distance to a facility, mi	NA	NA	NA	NA	−0.02 (−0.03 to −0.01)	.003

Abbreviation: NA, not applicable.

Legislative coefficients represent the change in the abortion rate associated with the enforcement of a highly restrictive legislative climate, which was defined as states having restrictions in 3 or more of 4 major categories of laws, compared with a less-restrictive climate. Totals represent number of county observations included in the model and differ based on availability of abortion rate and distance data. All models were difference-in-difference estimates and employed propensity score weighting, state and year fixed effects, and cluster-robust standard errors.

^a^ The distance coefficient represents the change in the abortion rate associated with each additional mile of distance to a facility providing abortion care.

**Table 4.** Change in Distance to a Facility Providing Abortion Care Associated With Highly Restrictive Legislative Climate

	Primary model (n = 24 896)	Only states that became highly restrictive (n = 20 304)	Only states that had abortion data (n = 5379)	Only states that had abortion data and became highly restrictive (n = 4504)
β (95% CI)	−2.73 (−6.02 to 0.57)	−3.24 (−7.10 to 0.62)	−6.78 (−19.39 to 5.83)	−7.68 (−21.45 to 6.09)
*P* value	.10	.10	.27	.25

Coefficients represent the change in distance to a facility providing abortion care (measured continuously in miles) associated with the enforcement of a highly restrictive legislative climate, defined by having restrictions in 3 or 4 major categories of laws, compared to a less-restrictive climate, with laws in 2 or fewer categories. Reported totals in the heading represent the number of county observations included in the model, and differ based on availability of abortion rate and distance data. All models are difference-in-difference estimates, and employ propensity score weighting, state and year fixed effects, and cluster-robust standard errors.

## Discussion

A growing body of evidence suggests that restrictive state legislation may pose a barrier to individuals seeking abortion. This study extends this literature by providing evidence from a multistate, longitudinal analysis that suggests that a highly restrictive state legislative climate is associated with a significantly lower abortion rate. With a median abortion rate of 2.89 abortions per 1000 women, a drop of 0.48 abortions per 1000 women associated with a highly restrictive policy climate represents a clinically meaningful 17% change in the abortion rate. The fact that we obtained concordant results from the model that included only states that became highly restrictive during the study period further supports our conclusion because this direct comparison between states with a highly restrictive policy and those in the prepolicy period during the same calendar year minimized the likelihood of confounding factors based on underlying differences between states that became highly restrictive and those that never did.

Moreover, while prior data from individual states suggest that restrictive laws may be associated with clinic closures and therefore with increased travel distances,^5,6,7^ we did not find this to be the case. In our study, a highly restrictive policy climate was not associated with an increased distance to a facility providing abortion care, but was associated with a lower abortion rate, suggesting that a restrictive climate itself may act as a barrier to abortion care.

Our findings provide additional context for Austin and Harper’s work^11^ on restrictive legislation. While their article, using a methodology similar to ours, did not identify a statistically significant effect of individual policies, our paper used a composite measure to evaluate overall restrictiveness of the legislative climate, with distance included as a covariate. While our data cannot elucidate the dynamics of how a restrictive policy climate may interfere with an individual’s attempts to access care, data such as those from Texas in the wake of HB2 have shown that even patients who ultimately reach a facility providing abortion care find restrictions increase the difficulty of seeking such care.^5^ Moreover, being denied abortion care is associated with increases in psychological stress, risk of pregnancy-related health complications, and financial instability.^39,40,41^ Together, these findings suggest that while the individual effect of a given policy may be difficult to identify, the cumulative effect of multiple laws that generate a restrictive legislative climate may pose a barrier to individuals who need abortions, resulting in harm.

Chief among this article’s strengths is the use of longitudinal data from a diverse set of states, thereby potentially improving external validity in comparison to single-state studies. Additionally, the difference-in-difference method provides robust (though not definitive) evidence that the change in the abortion rate identified is associated with imposition of restrictive policies and likely not other factors that may affect the abortion rate.

### Limitations

Our study does have several limitations. First, although the set of states included was diverse, there were differences between these included states and those that did not provide useable data. It is possible that our results would be different if additional states could have been included. That said, the absolute size of the differences for several variables was small, suggesting this may not have been a large source of bias in our study. Second, we were only able to generate abortion rates for all women rather than for those of reproductive age. Third, while we have a large longitudinal data set, we do not have data from more recent years, when a number of additional restrictive laws have been implemented. It is possible that our findings might change if we had access to more recent data. Fourth, while the results of our models of the association between restrictive policy climate and distance to a facility were nonsignificant, these point estimates were consistently negative. Further research may uncover additional nuances in the dynamic between policy and facility location.

## Conclusions

This study provides evidence that a highly restrictive state legislative climate is associated with a lower abortion rate. The methodology used suggests that this abortion rate drop is related to the imposition of restrictive policies rather than other factors that may drive the abortion rate. We conclude that the cumulative effect of restrictive policies may pose a barrier to patients accessing abortion care.
